# Bio-concentration potential and associations of heavy metals in *Amanita muscaria* (L.) Lam. from northern regions of Poland

**DOI:** 10.1007/s11356-018-2603-0

**Published:** 2018-06-25

**Authors:** Jerzy Falandysz, Małgorzata Mędyk, Roland Treu

**Affiliations:** 1 Laboratory of Environmental Chemistry & Ecotoxicology, Gdańsk University, 63 Wita Stwosza Street, 80-308 Gdańsk, Poland; 2 Faculty of Science and Technology, Athabasca University, 1 University Drive, Athabasca, AB T9S 3A3 Canada

**Keywords:** *Amanita muscaria*, Environment, Metallic contaminants, Bioconcentration, Forest, Fungi, Geochemical cycle

## Abstract

Fruiting bodies of *Amanita muscaria* and topsoil beneath from six background areas in northern regions of Poland were investigated for the concentration levels of Ag, Al, Ba, Ca, Cd, Co, Cu, Fe, Hg, K, Mg, Mn, Na, Rb, Sr, and Zn. In addition, the bioconcentration factors (BCF values) were studied for each of these metallic elements. Similar to studies from other basidiomycetes, *A. muscaria* showed species-specific affinities to some elements, resulting in their bioconcentration in mycelium and fruiting bodies. This mushroom growing in soils with different levels of the geogenic metallic elements (Ag, Al, Ba, Ca, Co, Cu, Fe, Hg, K, Mg, Mn, Na, Rb, Sr, and Zn) showed signs of homeostatic accumulation in fruiting bodies of several of these elements, while Cd appeared to be accumulated at a rate dependent of the concentration level in the soil substrate. This species is an efficient bio-concentrator of K, Mg, Cd, Cu, Hg, Rb, and Zn and hence also contributes to the natural cycling of these metallic elements in forest ecosystems.

## Introduction

*Amanita muscaria* (fly agaric) is a spectacular and well recognizable ectomycorrhizal mushroom that is native and common in the conifer and deciduous forests of the temperate and boreal zones of the northern hemisphere. In recent decades, it was introduced by forestry into the southern hemisphere and hence became a cosmopolitan species (Reid and Eicker 1991).

Fly agaric is widely known as psychoactive due to the hallucinogenic effects of some of its compounds. This mycorrhizal species is considered as specifically efficient for the bio-concentration of vanadium and cadmium in fruiting bodies (Drewnowska et al. 2013; Falandysz et al. 2007a, b; Lepp et al. 1987; Vetter 2005). *Amanita muscaria* is considered as a toadstool, i.e., an inedible or poisonous mushroom. A case of intoxication and coma of a young man after ingestion of *A. muscaria* was reported in one study from Poland (Mikaszewska-Sokolewicz et al. 2016). Due to its reputation, *A. muscaria* is usually avoided and rarely foraged; therefore, its fruiting bodies are abundant and available for research. Traditionally, it has also been used for catching flies (Lumpert and Kreft 2016). Little is known on the bio-concentration potential of heavy metals in *A. muscaria* across different soil types and geochemical variations and from areas with various amounts of pollution.

Ibotenic acid and muscimol are the two important psychoactive compounds in the fruiting bodies of *Amanita muscaria*. Muscimol is considered the principal psychoactive component and it is much more potent than ibotenic acid. After ingestion or drying of *A. muscaria* fruiting bodies, decarboxylases convert ibotenic acid ((S)-2-amino-2-(3-hydroxyisoxazol-5-yl) acetic acid) to muscimol. Both muscimol and ibotenic acid activate the neurotransmitter gamma-aminobutyric acid (GABA) receptors and *A. muscaria* can affect neuronal activity in the central regions of the brain (Michelot and Melendez-Howell 2003; Ogawa et al. 2015). While there is no doubt that the consumption of *A. muscaria* can lead to poisonings (Mikaszewska-Sokolewicz et al. 2016), the consumption of *A. muscaria* or its products for hallucinatory effects by the native Siberian shamans and possibly also in other locations in Eurasia was frequently not accompanied by symptoms of poisonings (Wasson 1968; http 2016). Therefore, the effect of treatment of the fruitbodies before consumption may be important (Feeney 2010). Other aspects of *A. muscaria* toxicology are discussed by Michelot and Melendez-Howell (2003), Tsujikawa et al. (2006), and by Vendramin and Brvar (2014).

Muscimol and ibotenic acid are well soluble in water—with a solubility of up to 100 mM for muscimol and of up to 10 mM for ibotenic acid (ChemSpider 2017). Blanching or parboiling of mushrooms is a common treatment before cooking: they are boiled in excess water for 10–15 min and the waste water is subsequently discarded (Falandysz and Drewnowska 2017). As reported by some authors, *Amanita muscaria* fruiting bodies (de-peeled caps and young stipes) sliced into small pieces and blanched (parboiled) for 15 min or blanched twice (for 5 and 5 min) with a large excess of water will lose their muscimol and ibotenic acid after the waste water is discarded. This treatment detoxifies the mushrooms from the hallucinogens and renders them well edible (Rubel and Arora 2008; Marley 2010).

It is worth to mention that blanching of mushroom’s fruiting bodies can lead also to partial but significant loss of some other organic constituents and numerous minerals during such preparation (Biekman et al. 1996; Drewnowska et al. 2017a, b; Falandysz and Drewnowska 2015 and 2017).

In the region of the Nagano prefecture in Japan, it is a tradition to preserve caps of the fruiting bodies of *A. muscaria* in salt after blanching (Phipps et al. 2000). Apart from Japan, there are also reports of culinary use of *A. muscaria* as pickles from Lithuania, Finland, Russia, and very recently from the North America (Rubel and Arora 2008).

The aim of this work was to assess the bioconcentration and bio-indication potential of *Amanita muscaria* fruiting bodies for the metallic elements Ag, Al, Ba, Ca, Cd, Co, Cu, Fe, Hg, K, Mg, Mn, Na, Rb, Sr, and Zn. Samples of fruiting bodies were collected together with the top layer of soil beneath. Six locations of forested or woodland regions from northern Poland were selected. These areas are not subject to direct impacts from local or regional heavy metals emitters. Forest soils in the northern part of Poland are slightly acidic and differ regionally and locally according to the differences in soil parent material; podzolic, brown, and rusty soils were formed where conifers dominate. In the region of Pomerania (Kaszuby and Kociewie region), poor sands with or without clay background (remains of a recent glacial epoch) are frequently dominant (DRRiP 2000). Planted scots pines (*Pinus silvestris*) and spruce (*Picea abies*) are predominant, with small enclaves of birch (*Betula* spp., often self-introduced) occasionally forming mixed stands. Locally, European larch (*Larix decidua*), European beech (*Fagus sylvatica*), or oak trees (*Quercus petraea* or *Quercus robur*) are planted. Older forests may include maples (*Acer platanoides*) and European hornbeam (*Carpinus betulus*), while *Alnus glutinosa*, the common alder, is frequent in wetlands.

## Materials and methods

**Fig. 1:**
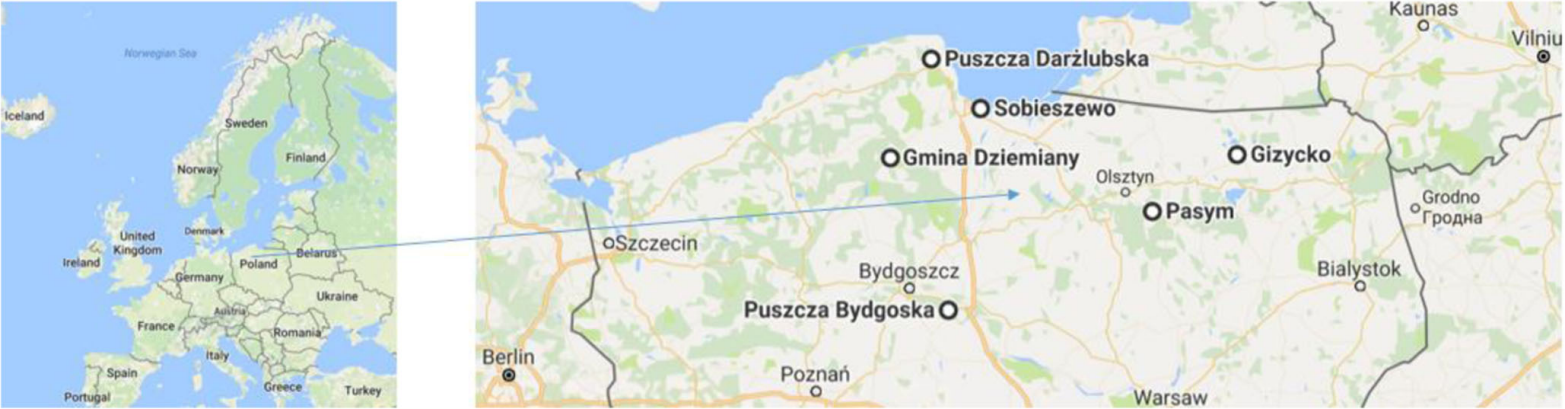
Sampling sites of *A. muscaria*: Sobiszewo (Sobieszewo Island), Puszcza Darżlubska (Darżlubska Wilderness), Gmina Dziemiany (Dziemiany), Puszcza Bydgoska (Bydgoska forests), Pasym, and Giżycko

Fruiting bodies of *A. muscaria* and the surface soil layer (0–10 cm) underneath the fruiting bodies were collected from six sites: Sobieszewo Island forest (N 53° 48′ 22″ E 18° 18′ 50″), Puszcza Darżlubska (Darżlubska Wilderness) in the vicinity of Wejherowo, forests in the County of Dziemiany, Bydgoska Wilderness in the Pomerania region, forests near the town of Pasym, and in the vicinity of Giżycko town in the Warmia and Masuria region of Poland (Fig. 1; Table 1). At each site 8–15 composite fruiting body samples were taken. Each sample consisted of 24–45 well-developed fruiting bodies. Caps and stipes were separated. Likewise, 8–15 composite topsoil samples were taken from underneath the fruiting bodies.

**Table 1:** Elements content of the caps (C) and stipes (S) of *Amanita muscaria* and beneath forest soil, and elements’ BCF values (mean ± SD, median value, and range)

Element	Matrix	Site					
		Sobieszewo Island *n* = 8 (24)^*^	Darżlubska Wilderness, Wejherowo *n* = 15 (44)	Kaszuby, Dziemiany *n* = 15 (23)	Bydgoska forests *n* = 15 (45)	Pasym, Warmia *n* = 15 (30)	Giżycko, Warmia *n* = 15 (28)
mg kg^−1^ dm							
K	Cap	39,000 ± 2000	38,000 ± 6000	50,000 ± 9000	43,000 ± 5000	34,000 ± 6000	40,000 ± 3000
		40,000	37,000	53,000	45,000	36,000	40,000
		36,000–41,000	29,000–49,000	31,000–61,000	35,000–51,000	25,000–45,000	34,000–46,000
	Stipe	32,000 ± 4000	24,000 ± 8000	38,000 ± 10,000	38,000 ± 4000	20,000 ± 5000	25,000 ± 6000
		31,000	25,000	38,000	38,000	19,000	26,000
		29,000–40,000	800–34,000	19,000–56,000	32,000–44,000	15,000–34,000	17,000–33,000
	Soil PT	540 ± 150	460 ± 110	800 ± 460	320 ± 170	580 ± 170	330 ± 20
		480	430	740	380	600	330
		440–810	310–630	290–1400	300–780	360–760	300–350
	Soil E	31 ± 2	85 ± 37	110 ± 60	43 ± 16	98 ± 49	38 ± 15
		31	86	110	43	91	45
		28–34	42–14	33–180	26–61	55–160	15–46
	^a^BCF_C-pt_	76 ± 15	120 ± 16	120 ± 120	140 ± 17	77 ± 47	120 ± 7
		82	120	95	150	83	120
		49–88	110–130	38–210	130–160	43–110	110–120
	^a^BCF_S-pt_	64 ± 18	77 ± 3	66 ± 55	110 ± 12	37 ± 16	70 ± 15
		69	77	69	110	34	69
		39–83	75–80	27–100	100–120	26–48	59–80
	^b^BCF_C-e_	1200 ± 59	740 ± 340	1100 ± 1100	800 ± 95	570 ± 220	1800 ± 1200
		1200	720	1400	820	590	1300
		1200–1300	500–980	290–1800	740–870	410–720	900–2600
	^b^BCF_S-e_	1100 ± 140	470 ± 180	560 ± 500	610 ± 10	280 ± 48	1100 ± 980
		980	480	350	610	280	1200
		920–1200	340–590	210–920	600–620	250–320	450–1800
Mg	Cap	920 ± 60	870 ± 10	850 ± 150	1100 ± 100	980 ± 100	1100 ± 100
		920	850	880	1100	1000	1100
		850–1000	750–1100	580–1200	910–1300	780–1100	1000–1300
	Stipe	560 ± 480	490 ± 70	460 ± 90	610 ± 40	530 ± 110	490 ± 90
		560	490	440	600	530	500
		500–620	370–580	300–610	570–690	390–690	340–670
	Soil PT	590 ± 0.340	720 ± 270	1000 ± 100	390 ± 10	1200 ± 400	540 ± 20
		450	730	1100	400	1200	540
		410–1200	410–1100	850–1200	370–410	780–1900	500–560
	Soil E	110 ± 10	300 ± 140	350 ± 120	160 ± 40	370 ± 190	90 ± 14
		100	320	330	160	350	92
		90–120	130–500	220–510	120–200	170–620	71–110
	BCF_C-pt_	1.9 ± 0.6	1.7 ± 0.5	0.98 ± 0.73	2.7 ± 0.2	0.97 ± 0.39	2.1 ± 0.1
		2.0	1.4	0.65	2.7	0.82	2.1
		0.76–2.3	1.4–2.1	0.46–1.5	2.5–2.7	0.69–1.2	2.0–2.3
	BCF_S-pt_	1.1 ± 0.4	0.95 ± 0.31	0.41 ± 0.28	1.5 ± 0.1	0.55 ± 0.07	1.1 ± 0.2
		1.3	0.88	0.47	1.5	0.55	1.0
		0.45–1.5	0.73–1.2	0.21–0.61	1.5–1.6	0.50–0.60	0.95–1.2
	BCF_C-e_	8.8 ± 1.2	4.6 ± 1.8	4.2 ± 3.9	5.7 ± 0.6	3.5 ± 0.1	13 ± 0
		8.5	5.0	2.6	5.7	3.5	13
		7.6–10	3.3–5.9	1.4–6.9	5.2–6.1	3.5–3.5	13–13
	BCF_S-e_	5.3 ± 0.6	2.5 ± 1.1	1.7 ± 1.6	3.2 ± 0.4	2.2 ± 1.1	6.4 ± 1.1
		5.0	2.4	1.2	3.3	2.3	6.8
		4.8–6.0	1.8–3.3	0.62–2.8	3.0–3.5	1.4–3.0	5.6–7.2
mg kg ^−1^ dm							
Ag	Cap	0.70 ± 0.09	0.50 ± 0.10	3.9 ± 1.4	0.81 ± 0.16	0.72 ± 0.13	0.72 ± 0.18
		0.72	0.48	3.3	0.81	0.70	0.70
		0.55–0.79	0.39–0.74	2.3–6.4	0.68–1.1	0.54–0.89	0.43–1.0
	Stipe	0.78 ± 0.33	0.63 ± 0.21	1.4 ± 0.6	0.86 ± 0.11	0.44 ± 0.10	0.53 ± 0.30
		0.72	0.57	1.2	0.86	0.48	0.55
		0.45–1.3	0.34–1.2	0.77–2.5	0.71–1.1	0.27–0.57	0.16–1.0
	Soil PT	WD	WD	5.4 ± 3.0	1.8 ± 0.3	2.5 ± 1.5	1.5 ± 0.3
				5.0	1.8	3.0	1.4
				2.2–9.3	1.7–2.2	0.44–3.6	1.2–1.9
	BCF_C-pt_	WD	WD	1.6 ± 1.9	0.49 ± 0.03	0.85 ± 0.90	0.53 ± 0.09
				1.8	0.49	0.87	0.53
				0.27–2.9	0.46–0.50	0.21–1.5	0.46–0.59
	BCF_S-pt_	WD	WD	0.33 ± 0.34	0.52 ± 0.03	0.47 ± 0.45	0.39 ± 0.24
				0.38	0.52	0.50	0.45
				0.09–0.57	0.49–0.54	0.15–0.79	0.22–0.57
Al	Cap	490 ± 390	270 ± 160	190 ± 140	420 ± 200	170 ± 82	110 ± 140
		300	290	140	380	180	68
		210–1300	48–590	74–540	110–780	69–320	43–510
	Stipe	48 ± 47	210 ± 100	200 ± 110	200 ± 68	110 ± 35	58 ± 33
		34	220	160	200	110	61
		7.0–140	88–400	64–410	83–350	61–170	18–100
	Soil PT	5900 ± 4200	16,000 ± 2700	26,000 ± 3800	8600 ± 7200	18,000 ± 4500	14,000 ± 960
		4000	15,000	27,000	8500	17,000	14,000
		3800–13,000	12,000–20,000	20,000–30,000	7800–9600	14,000–25,000	13,000–15,000
	Soil E	1100 ± 42	6600 ± 2600	6700 ± 1400	2600 ± 810	5200 ± 1900	3400 ± 210
		1100	6700	6200	2400	5300	3400
		1000–1100	3400–10,000	5800–8700	1800–3700	3200–6900	3200–3600
	BCF_C-pt_	0.082 ± 0.051	0.015 ± 0.010	0.004 ± 0.001	0.039 ± 0.004	0.008 ± 0.006	0.006 ± 0.001
		0.069	0.014	0.004	0.039	0.007	0.006
		0.023–0.15	0.008–0.022	0.004–0.005	0.036–0.042	0.003–0.012	0.006–0.007
	BCF_S-pt_	0.009 ± 0.007	0.009 ± 0.004	0.013 ± 0.002	0.030 ± 0.014	0.006 ± 0.001	0.005 ± 0.001
		0.008	0.009	0.013	0.028	0.006	0.005
		0.001–0.019	0.006–0.012	0.011–0.014	0.020–0.041	0.005–0.007	0.004–0.006
	BCF_C-e_	0.35 ± 0.16	0.027 ± 0.019	0.036 ± 0.023	0.14 ± 0.03	0.021 ± 0.013	0.027 ± 0.006
		0.26	0.022	0.042	0.14	0.023	0.028
		0.22–0.59	0.014–0.040	0.020–0.052	0.12–0.16	0.012–0.031	0.023–0.031
	BCF_S-e_	0.034 ± 0.023	0.016 ± 0.007	0.033 ± 0.017	0.11 ± 0.06	0.018 ± 0.001	0.023 ± 0.007
		0.030	0.014	0.035	0.09	0.018	0.022
		0.009–0.067	0.011–0.021	0.021–0.045	0.07–0.16	0.017–0.018	0.018–0.028
Ba	Cap	2.0 ± 1.0	1.4 ± 0.4	1.2 ± 0.6	1.8 ± 0.9	0.72 ± 0.27	0.88 ± 0.77
		1.6	1.2	1.2	1.6	0.69	0.51
		1.2–4.2	0.99–2.0	0.37–2.7	0.61–3.6	0.36–1.3	0.41–2.9
	Stipe	0.50 ± 0.40	1.5 ± 0.2	1.4 ± 0.7	1.1 ± 0.4	0.69 ± 0.27	0.73 ± 0.38
		0.40	1.6	1.3	1.3	0..66	0.75
		0.10–1.3	1.2–2.0	0.63–3.0	0.45–1.6	0.35–1.2	0.23–1.3
	Soil PT	73 ± 54	160 ± 45	130 ± 15	87 ± 14	120 ± 28	67 ± 5
		49	150	130	90	130	67
		45–170	94–220	120–150	69–99	89–160	60–72
	Soil E	41 ± 4	120 ± 28	86 ± 4	76 ± 17	110 ± 21	35 ± 2
		41	120	86	78	110	34
		37–47	78–160	82–91	56–92	80–130	33–37
	BCF_C-pt_	0.031 ± 0.014	0.013 ± 0.002	0.014 ± 0.013	0.013 ± 0.002	0.006 ± 0.001	0.019 ± 0.001
		0.035	0.013	0.018	0.013	0.006	0.019
		0.009–0.043	0.012–0.015	0.005–0.023	0.012–0.014	0.005–0.007	0.018–0.020
	BCF_S-pt_	0.008 ± 0.005	0.014 ± 0.001	0.017 ± 0.012	0.015 ± 0.003	0.006 ± 0.001	0.014 ± 0.004
		0.008	0.015	0.013	0.015	0.006	0.016
		0.002–0.014	0.014–0.015	0.009–0.026	0.013–0.017	0.005–0.006	0.011–0.016
	BCF_C-e_	0.042 ± 0.010	0.015 ± 0.001	0.021 ± 0.017	0.014 ± 0.002	0.006 ± 0.001	0.039 ± 0.001
		0.037	0.015	0.036	0.014	0.006	0.039
		0.032–0.057	0.014–0.16	0.010–0.033	0.013–0.015	0.005–0.007	0.038–0.039
	BCF_S-e_	0.010 ± 0.005	0.017 ± 0.001	0.026 ± 0.015	0.016 ± 0.003	0.006 ± 0.001	0.027 ± 0.007
		0.010	0.017	0.024	0.016	0.006	0.026
		0.004–0.016	0.016–0.017	0.016–0.037	0.014–0.018	0.006–0.007	0.022–0.032
Ca	Cap	99 ± 49	150 ± 50	120 ± 61	160 ± 60	110 ± 38	98 ± 52
		99	160	84	130	110	87
		41–180	75–220	48–240	92–250	54–170	39–240
	Stipe	76 ± 48	240 ± 71	120 ± 67	200 ± 79	120 ± 71	99 ± 55
		77	260	120	220	100	95
		34–170	110–330	41–230	79–330	48–290	39–220
	Soil PT	640 ± 340	1800 ± 1400	510 ± 110	380 ± 130	810 ± 740	200 ± 27
		490	1400	520	390	720	190
		380–1200	600–4400	360–640	240–490	180–1600	170–230
	Soil E	530 ± 65	1700 ± 1000	250 ± 170	310 ± 220	740 ± 500	44 ± 10
		500	1500	230	320	690	41
		460–610	620–3400	81–460	54–530	270–1300	37–59
	BCF_C-pt_	0.18 ± 0.09	0.25 ± 0.13	0.27 ± 0.19	0.25 ± 0.01	0.51 ± 0.15	0.46 ± 0.10
		0.20	0.26	0.28	0.25	0.55	0.46
		0.04–0.29	0.15–0.34	0.13–0.40	0.24–0.26	0.40–0.62	0.38–0.53
	BCF_S-pt_	0.12 ± 0.07	0.33 ± 0.12	0.29 ± 0.21	0.44 ± 0.01	0.63 ± 0.18	0.50 ± 0.13
		0.11	0.32	0.24	0.44	0.61	0.51
		0.03–0.19	0.25–0.42	0.14–0.44	0.43–0.44	0.50–0.80	0.41–0.59
	BCF_C-e_	0.18 ± 0.07	0.27 ± 0.12	1.4 ± 1.7	0.25 ± 0.01	0.89 ± 0.06	2.1 ± 0.8
		0.17	0.28	1.3	0.25	0.89	2.2
		0.09–0.27	0.18–0.35	0.18–2.6	0.24–0.26	0.84–0.93	1.5–2.7
	BCF_S-e_	0.13 ± 0.05	0.37 ± 0.09	1.5 ± 1.8	0.44 ± 0.05	1.2 ± 0.6	2.2 ± 0.2
		0.16	0.36	1.4	0.44	1.2	2.3
		0.06–0.18	0.30–0.43	0.20–2.8	0.41–0.47	0.76–1.6	2.0–2.4
Cd	Cap	19 ± 10	10 ± 3	15 ± 6	21 ± 5	14 ± 5	13 ± 7
		13	10	15	21	14	11
		9.0–36	5.7–14	4.0–25	9.0–25	7.0–26	6.0–32
	Stipe	10 ± 6	4.2 ± 1.7	8.7 ± 5.5	10 ± 4	5.6 ± 2.2	7.5 ± 7.4
		6.0	4.2	7.4	11	4.9	3.6
		5.0–22	2.0–7.1	1.4–20	4.0–17	3.4–10	2.1–27
	Soil PT	0.036 ± 0.027	0.098 ± 0.062	0.049 ± 0.013	0.052 ± 0.017	0.050 ± 0.017	0.014 ± 0.003
		0.026	0.086	0.046	0.055	0.049	0.013
		0.017–0.083	0.035–0.21	0.036–0.066	0.029–0.068	0.034–0.068	0.012–0.018
	Soil E	0.025 ± 0.001	0.097 ± 0.036	0.047 ± 0.016	0.034 ± 0.011	0.036 ± 0.009	0.005 ± 0.003
		0.025	0.10	0.049	0.019	0.037	0.006
		0.024–0.026	0.049–0.14	0.028–0.061	0.029–0.050	0.025–0.048	0.002–0.008
	BCF_C-pt_	840 ± 580	200 ± 17	330 ± 6	440 ± 19	290 ± 140	1100 ± 440
		280	200	330	440	240	1300
		120–900	180–210	320–330	430–450	190–390	830–1400
	BCF_S-pt_	440 ± 340	94 ± 12	130 ± 16	240 ± 11	110 ± 18	530 ± 260
		280	91	130	240	110	510
		120–900	86–100	120–150	230–250	99–120	340–710
	BCF_C-e_	890 ± 420	270 ± 30	350 ± 200	840 ± 78	360 ± 240	2900 ± 870
		990	270	400	810	380	2700
		450–1400	250–290	210–500	770–490	190–530	2300–3500
	BCF_S-e_	460 ± 270	130 ± 13	150 ± 90	460 ± 27	130 ± 8	1300 ± 220
		410	130	180	460	130	1200
		210–850	120–140	83–210	420–490	120–140	1100–1400
Co	Cap	0.32 ± 0.06	0.16 ± 0.04	0.20 ± 0.15	0.34 ± 0.10	0.25 ± 0.03	0.51 ± 0.23
		0.31	0.15	0.19	0.30	0.25	0.44
		0.25–0.40	0.10–0.24	0.04–0.54	0.24–0.61	0.22–0.31	0.29–1.1
	Stipe	0.36 ± 0.05	0.15 ± 0.02	0.16 ± 0.12	0.28 ± 0.08	0.24 ± 0.03	0.40 ± 0.15
		0.35	0.14	0.13	0.26	0.24	0.37
		0.29–0.45	0.12–0.19	0.03–0.42	0.22–0.53	0.19–0.29	0.23–0.78
	Soil PT	0.56 ± 0.36	0.91 ± 0.25	1.4 ± 0.5	0.39 ± 0.04	0.86 ± 0.10	0.45 ± 0.02
		0.40	0.86	1.5	0.39	0.84	0.45
		0.37–1.2	0.59–1.3	0.78–2.1	0.35–0.44	0.77–0.99	0.42–0.47
	Soil E	0.22 ± 0.01	0.53 ± 0.24	1.1 ± 0.5	0.28 ± 0.10	0.60 ± 0.05	0.16 ± 0.01
		0.23	0.47	1.2	0.24	0.58	0.45
		0.21–0.23	0.30–0.90	0.49–1.7	0.22–0.43	0.57–0.67	0.42–0.47
	BCF_C-pt_	0.62 ± 0.17	0.20 ± 0.04	0.31 ± 0.26	0.76 ± 0.07	0.26 ± 0.05	1.6 ± 1.1
		0.69	0.20	0.32	0.76	0.26	1.7
		0.32–0.74	0.18–0.23	0.13–0.49	0.72–0.81	0.23–0.30	0.83–2.4
	BCF_S-pt_	0.76 ± 0.28	0.20 ± 0.04	0.30 ± 0.27	0.70 ± 0.12	0.24 ± 0.01	1.1 ± 0.9
		0.80	0.20	0.27	0.71	0.24	1.0
		0.31–1.0	0.17–0.22	0.11–0.49	0.62–0.79	0.23–0.25	0.50–1.8
	BCF_C-e_	1.3 ± 0.2	0.33 ± 0.05	0.47 ± 0.44	1.2 ± 0.1	0.39 ± 0.01	4.6 ± 2.8
		1.2	0.33	0.58	1.2	0.39	4.8
		1.2–1.6	0.29–0.37	0.15–0.78	1.2–1.3	0.38–0.40	2.6–6.6
	BCF_S-e_	1.6 ± 0.2	0.32 ± 0.05	0.45 ± 0.46	1.1 ± 0.0	0.37 ± 0.04	3.2 ± 2.3
		1.5	0.32	0.51	1.1	0.37	3.0
		1.4–1.9	0.28–0.35	0.12–0.78	1.1–1.1	0.34–0.39	1.5–4.9
Cu	Cap	50 ± 19	35 ± 15	39 ± 11	40 ± 4	41 ± 8	33 ± 11
		48	30	37	39	43	30
		25–85	19–69	22–62	36–50	28–53	18–52
	Stipe	27 ± 12	10 ± 2	16 ± 5	17 ± 4	15 ± 4	14 ± 4
		24	10	17	15	14	14
		13–50	6.6–14	8.0–24	13–23	10–20	7.9–20
	Soil PT	1.0 ± 1.1	1.9 ± 0.9	4.0 ± 2.3	1.0 ± 0.5	1.6 ± 0.5	0.86 ± 0.11
		0.52	1.8	4.4	1.1	1.5	0.89
		0.33–3.0	0.76–3.1	0.86–6.2	0.35–1.6	1.1–2.3	0.69–0.95
	Soil E	0.32 ± 0.03	1.0 ± 0.4	2.3 ± 1.6	0.78 ± 0.38	1.4 ± 1.4	0.36 ± .0.03
		0.31	0.98	2.3	0.84	0.82	0.35
		0.29–0.37	0.46–1.5	0.37–4.3	0.30–1.1	0.67–3.5	0.33–0.41
	BCF_C-pt_	110 ± 73	46 ± 10	39 ± 47	34 ± 1	27 ± 6	43 ± 2
		130	41	18	34	27	43
		15–190	39–53	6.0–72	34–35	23–32	41–45
	BCF_S-pt_	56 ± 38	11 ± 0	11 ± 12	13 ± 1	12 ± 1	16 ± 1
		61	11	7.0	13	12	16
		7.0–98	11–11	2.0–20	13–14	11–13	15–16
	BCF_C-e_	180 ± 38	76 ± 18	90 ± 11	67 ± 62	53 ± 1	110 ± 3
		160	72	76	84	53	110
		130–230	63–89	11–170	23–110	53–54	110–120
	BCF_S-e_	90 ± 27	18 ± 1	25 ± 30	26 ± 24	24 ± 8	42 ± 1
		79	18	28	38	28	42
		66–140	17–18	4.0–46	9.0–43	18–30	42–43
Fe	Cap	310 ± 400	140 ± 68	130 ± 79	240 ± 97	74 ± 31	180 ± 160
		170	120	100	230	65	110
		49–800	33–250	60–320	85–430	37–140	77–610
	Stipe	83 ± 93	110 ± 56	130 ± 62	110 ± 29	51 ± 13	79 ± 53
		60	97	110	110	54	74
		13–280	48–220	54–260	56–150	35–66	26–220
	Soil PT	2500 ± 600	2800 ± 560	3500 ± 850	1000 ± 680	2600 ± 620	2100 ± 94
		2200	3000	3400	1200	2600	2000
		2000–3500	2000–3400	2600–4600	1000–1500	1900–3400	2000–2200
	Soil E	850 ± 32	1400 ± 550	1900 ± 310	620 ± 150	1400 ± 430	1100 ± 120
		860	1300	1900	620	1400	1100
		800–890	540–2200	1700–2400	450–790	920–1800	990–1300
	BCF_C-pt_	0.080 ± 0.057	0.044 ± 0.025	0.062 ± 0.063	0.32 ± 0.07	0.020 ± 0.012	0.084 ± 0.018
		0.078	0.042	0.019	0.34	0.023	0.084
		0.027–0.17	0.026–0.062	0.017–0.11	0.28–0.37	0.012–0.029	0.072–0.097
	BCF_S-pt_	0.026 ± 0.020	0.028 ± 0.010	0.060 ± 0.056	0.21 ± 0.06	0.016 ± 0.006	0.074 ± 0.034
		0.028	0.029	0.037	0.23	0.016	0.075
		0.007–0.057	0.021–0.035	0.021–0.10	0.17–0.26	0.012–0.021	0.050–0.099
	BCF_C-e_	0.22 ± 0.13	0.065 ± 0.034	0.086 ± 0.073	WD	0.034 ± 0.015	0.17 ± 0.01
		0.21	0.054	0.061		0.039	0.17
		0.08–0.43	0.041–0.090	0.034–0.14		0.023–0.045	0.16–0.18
	BCF_S-e_	0.069 ± 0.045	0.041 ± 0.012	0.085 ± 0.063	WD	0.028 ± 0.007	0.16 ± 0.09
		0.074	0.041	0.052		0.026	0.18
		0.020–0.14	0.033–0.050	0.041–0.13		0.023–0.033	0.09–0.22
Hg	Cap	0.76	0.40	0.22	0.45	0.75	0.68
	Stipe	0.45	0.24	0.21	0.23	0.45	0.35
	Soil	0.015	0.054	0.010	0.013	0.031	0.031
	BCF_C_	49	17	19	51	29	22
	BCF_S_	28	10	18	23	15	19
Mn	Cap	8.3 ± 2.5	8.6 ± 3.7	14 ± 6	17 ± 4	11 ± 3	11 ± 6
		7.6	8.6	14	18	10	9.4
		6.1–12	5.1–19	6.0–24	10–23	8.9–18	6.2–29
	Stipe	6.0 ± 3	7.3 ± 2.5	14 ± 7	14 ± 6	10 ± 5	13 ± 8
		4.5	6.3	12	12	8.0	11
		4.1–12	3.9–12	6.0–29	7.0–25	4.7–22	4.8–29
	Soil PT	37 ± 29	100 ± 60	86 ± 11	82 ± 44	74 ± 9	23 ± 2
		23	76	84	84	76	23
		22–89	60–200	74–100	37–120	63–83	20–26
	Soil E	22 ± 1	100 ± 46	88 ± 15	45 ± 38	71 ± 11	13 ± 1
		22	97	84	44	72	13
		21–23	53–180	75–110	10–94	56–82	12–15
	BCF_C-pt_	0.29 ± 0.12	0.18 ± 0.11	0.18 ± 0.07	0.21 ± 0.07	0.13 ± 0.00	0.54 ± 0.08
		0.31	0.13	0.17	0.23	0.13	0.56
		0.08–0.41	0.10–0.26	0.14–0.23	0.15–0.26	0.13–0.13	0.48–0.59
	BCF_S-pt_	0.22 ± 0.14	0.11 ± 0.02	0.18 ± 0.10	0.27 ± 0.12	0.17 ± 0.14	0.51 ± 0.01
		0.20	0.11	0.14	0.33	0.12	0.51
		0.05–0.44	0.09–0.13	0.11–0.25	0.19–0.36	0.07–0.27	0.50–0.52
	BCF_C-e_	0.36 ± 0.08	0.20 ± 0.12	0.18 ± 0.06	WD	0.13 ± 0.00	1.0 ± 0.0
		0.34	0.24	0.18		0.13	1.0
		0.27–0.49	0.11–0.29	0.14–0.23		0.13–0.13	1.0–1.0
	BCF_S-e_	0.27 ± 0.14	0.12 ± 0.02	0.18 ± 0.09	WD	0.17 ± 0.14	0.99 ± 0.18
		0.21	0.12	0.14		0.12	0.91
		0.18–0.52	0.11–0.14	0.12–0.25		0.07–0.27	0.86–1.1
Na	Cap	59 ± 12	230 ± 500	53 ± 43	30 ± 7	390 ± 490	120 ± 200
		63	47	31	32	110	37
		39–76	23–1700	10–150	19–41	53–1500	16–670
	Stipe	100 ± 36	730 ± 1400	130 ± 210	92 ± 39	710 ± 730	750 ± 940
		110	270	41	100	350	370
		54–150	49–5000	21–640	23–150	130–2100	23–2600
	Soil PT	16 ± 11	34 ± 12	24 ± 4	12 ± 1	20 ± 4	15 ± 1
		12	31	23	12	22	15
		10–35	20–53	20–30	11–14	15–24	14–16
	Soil E	6.2 ± 2.8	23 ± 7	13 ± 7	8.6 ± 2.3	14 ± 3	16 ± 13
		5.8	24	11	8.7	13	11
		3.0–10	12–30	7.7–23	5.8–11	12–18	8.0–36
	BCF_C-pt_	4.6 ± 1.9	1.6 ± 0.4	3.2 ± 1.6	1.8 ± 0.4	18 ± 18	6.4 ± 1.8
		5.0	1.6	3.2	1.8	9.0	6.0
		1.4–6.3	1.3–1.9	2.1–4.4	1.5–2.1	5.0–31	5.2–7.7
	BCF_S-pt_	8.2 ± 3.8	4.2 ± 3.6	0.68 ± 0.52	8.0 ± 0.1	42 ± 34	40 ± 3
		9.2	2.5	0.98	8.0	27	40
		2.3–11	1.7–6.8	0.32–1.5	8.0–8.1	18–67	39–42
	BCF_C-e_	11 ± 6	1.7 ± 0.4	8.9 ± 3.9	2.1 ± 0.3	34 ± 40	7.5 ± 5.4
		9.1	1.7	8.4	2.2	48	7.9
		4.8–21	1.4–2.0	6.1–12	1.9–2.3	6.0–62	3.7–11
	BCF_S-e_	21 ± 12	4.4 ± 3.8	1.8 ± 1.3	9.5 ± 0.8	77 ± 80	56 ± 53
		19	4.1	1.5	9.4	69	58
		8.0–37	1.8–7.1	0.9–2.8	8.9–10	20–130	19–93
Rb	Cap	220 ± 56	340 ± 270	320 ± 88	140 ± 50	500 ± 170	620 ± 390
		210	260	310	110	470	470
		120–300	170–1100	240–420	80–210	320–820	270–1500
	Stipe	93 ± 36	88 ± 21	160 ± 89	66 ± 14	130 ± 71	280 ± 110
		86	88	110	66	110	310
		58–160	53–110	45–290	47–100	57–320	140–480
	Soil PT	2.3 ± 1.3	4.1 ± 1.0	1.1 ± 0.2	1.6 ± 1.0	3.9 ± 0.9	2.2 ± 0.1
		1.7	4.4	1.2	2.0	4.0	2.3
		1.5–4.7	2.7–5.2	0.88–1.4	0.02–2.3	2.7–5.0	2.0–2.4
	Soil E	0.26 ± 0.02	1.1 ± 0.7	0.86 ± 0.35	0.69 ± 0.19	0.90 ± 0.22	0.26 ± 0.15
		0.27	1.0	0.94	0.66	0.90	0.27
		0.23–0.28	0.21–2.0	0.31–1.2	0.49–0.94	0.66–1.1	0.22–0.30
	BCF_C-pt_	130 ± 52	180 ± 110	190 ± 45	230 ± 65	150 ± 74	320 ± 280
		130	130	180	220	130	390
		45–180	100–260	160–220	180–270	100–210	130–540
	BCF_S-pt_	57 ± 28	34 ± 11	97 ± 21	92 ± 4	35 ± 5	120 ± 60
		51	39	87	92	35	120
		18–94	26–42	72–110	89–95	32–39	80–160
	BCF_C-e_	930 ± 120	610 ± 340	620 ± 110	4400 ± 6200	670 ± 72	3000 ± 2200
		930	670	590	2500	680	2600
		760–1100	370–850	490–700	57–8800	620–720	1500–4600
	BCF_S-e_	400 ± 100	120 ± 50	130 ± 32	1500 ± 2100	160 ± 45	1100 ± 380
		370	110	120	990	150	1200
		310–580	87–160	190 ± 45	28–3100	130–190	880–1400
Sr	Cap	0.68 ± 0.36	0.45 ± 0.15	0.41 ± 019	0.70 ± 0.27	0.30 ± 0.12	0.24 ± 0.20
		0.56	0.40	0.36	0.69	0.28	0.19
		0.35–1.4	0.26–0.75	0.15–0.98	0.33–1.2	0.14–0.58	0.12–0.82
	Stipe	0.22 ± .0.18	0.48 ± 0.09	0.41 ± 0.19	0.65 ± 0.23	0.27 ± 0.11	0.27 ± 0.16
		0.20	0.47	0.37	0.68	0.26	0.26
		0.03–0.55	0.35–0.63	0.21–0.88	0.30–1.0	0.12–0.46	0.08–0.59
	Soil PT	19 ± 8	35 ± 10	34 ± 10	14 ± 10	26 ± 6	18 ± 1
		15	34	34	17	28	18
		15–33	23–51	23–44	1.0–22	17–31	17–19
	Soil E	7.6 ± 0.5	17 ± 6	8.2 ± 5.3	7.4 ± 5.5	11 ± 8	16 ± 1
		7.4	17	8.8	8.1	11	16
		7.1–8.2	9.0–25	2.3–13	0.97–12	3.9–19	15–18
	BCF_C-pt_	0.035 ± 0.012	0.017 ± 0.008	0.024 ± 0.025	0.047 ± 0.001	0.013 ± 0.004	0.012 ± 0.002
		0.037	0.019	0.036	0.047	0.013	0.012
		0.016–0.048	0.011–0.023	0.007–0.042	0.046–0.047	0.010–0.016	0.011–0.013
	BCF_S-pt_	0.011 ± 0.007	0.017 ± 0.001	0.023 ± 0.022	0.065 ± 0.001	0.013 ± 0.005	0.017 ± 0.005
		0.012	0.017	0.025	0.065	0.013	0.017
		0.004–0.021	0.017–0.018	0.007–0.038	0.065–0.066	0.009–0.017	0.013–0.021
	BCF_C-e_	0.079 ± 0.022	0.035 ± 0.008	0.23 ± 0.29	WD	0.071 ± 0.055	0.14 ± 0.03
		0.079	0.035	0.27		0.071	0.14
		0.052–0.11	0.030–0.041	0.02–0.43		0.069–0.072	0.11–0.16
	BCF_S-e_	0.026 ± 0.014	0.038 ± 0.010	0.21 ± 0.26	WD	0.079 ± 0.055	0.19 ± 0.03
		0.027	0.033	0.26		0.088	0.19
		0.009–0.042	0.031–0.045	0.02–0.39		0.040–0.12	0.17–0.22
Zn	Cap	140 ± 31	96 ± 25	140 ± 37	180 ± 27	120 ± 30	150 ± 35
		140	89	140	180	130	130
		120–190	65–150	86–200	140–240	71–170	99–200
	Stipe	91 ± 21	56 ± 10	82 ± 25	130 ± 27	73 ± 13	83 ± 23
		95	52	81	130	73	80
		66–120	45–73	46–140	95–193	57–93	49–130
	Soil PT	14 ± 11	16 ± 6	14 ± 2	7.2 ± 4.1	15 ± 3	6.2 ± 0.5
		8.0	15	14	7.4	15	6.2
		6.0–31	9.0–25	12–15	2.6–12	12–19	5.5–6.7
	Soil E	7.3 ± 0.6	10 ± 3	8.2 ± 1.8	5.6 ± 4.2	15 ± 5	2.6 ± 0.2
		7.2	12	8.3	5.3	16	2.6
		6.5–8.3	6.0–13	6.0–10	0.80–11	8.0–19	2.4–2.9
	BCF_C-pt_	16 ± 8	10 ± 2	13 ± 0	18 ± 4	8.8 ± 5.4	21 ± 5
		20	10	13	18	8.3	23
		4.0–24	9.0–12	13–13	16–21	5.0–13	17–25
	BCF_S-pt_	9.7 ± 5.6	5.3 ± 0.1	6.2 ± 0.2	12 ± 3	5.1 ± 1.4	13 ± 2
		11	5.3	6.2	12	5.8	13
		2.2–15	5.2–5.4	6.1–6.3	11–14	4.1–6.1	12–15
	BCF_C-e_	22 ± 5	15 ± 12	23 ± 2	120 ± 150	11 ± 10	54 ± 6
		19	12	23	49	13	53
		17–30	7.0–24	22–25	19–230	4.0–18	50–58
	BCF_S-e_	13 ± 1	7.3 ± 4.6	11 ± 1	85 ± 100	6.0 ± 3.8	34 ± 0
		11	7.4	11	29	6.4	34
		9.0–17	4.1–11	11–12	13–160	3.3–8.7	34–34

* Pooled samples (nubmer of individuals is given in parentheses). PT pseudo total, *E* extractable or leachable, *WD* without data. ^a^BCF_C-pt_ and ^a^BCF_S-pt_ (values of BCF respectively for caps and stipes calculated using data for the “pseudo-total” fraction of element in soil), ^b^BCF_C-e_ and ^b^BCF_S-e_ (values of BCF respectively for caps and stipes calculated using data for an extractable-leachable fraction of element in soil)

After collecting, mushrooms were cleaned up from debris of the litter and soil substrate. For each sample, caps and stipes (bottom part cut off) were separated from the fruiting bodies, sliced with a plastic knife, and dried at 65 °C for 24 h to constant mass using a commercial vegetable dehydrator with plastic trays (Falandysz et al. 2015). Dried caps and stipes were ground into a fine powder using a porcelain mortar, kept in air tight polyethylene bags, and stored under dry conditions.

Subsamples of 0.2–0.3 g of the fungal material were weighed into polytetrafluoroethylene (PTFE) pressure vessels and digested with 5 mL of concentrated nitric acid (HNO_3_) (Suprapure, Merck). After initial pre-digestion at room temperature and normal pressure for 24 h in semi-closed vessels, further digestion of the fungal material was achieved under pressure in an automatic digestion system (MLS 1200) in a microwave oven, and elements were determined by inductively coupled plasma-optical emission spectrometry (ICP-OES; Optima 2000 DV, Perkin-Elmer) with external standards using yttrium (20 mg L^−1^) (Brzostowski et al., 2011a, b). Mercury was determined by a validated method of cold vapor atomic absorption spectroscopy (CV-AAS) (Falandysz 1990).

Soil substratum samples (0–10 cm layer) underneath fruiting bodies were collected using plastic tools (knives and spoons) and were packed into sealed polyethylene bags. To obtain air-dried samples, opened polyethylene bags were covered with sheets of paper towels, kept in vertical position, and air-dried at room temperature for 16–18 weeks in the laboratory. Further, the soil samples were sieved through a 2-mm pore size plastic sieve and then stored until analysis for 4 to 8 weeks in brand new sealed polyethylene bags in a closed plastic box under dry conditions.

Before chemical analyses, topsoil samples were dried in an electric oven at 40 °C for 48 h. The extractable (labile) metallic elements from the soil samples (1.5 g) were extracted using 10 mL of 20% nitric acid solution (Suprapure, Merck) in open PTFE vessels that were gently heated up to 105 °C for 2 h (Kučak and Blanuša 1998). The pseudo-total metallic elements from the soil samples (1.0 g) were initially cold digested with 15 mL of concentrated nitric acid solution (65%, analytical grade; Suprapure, Merck) in Pyrex glass round bottom flasks for 16 h and further were hot digested for 2.5 h (Sastre et al. 2002). Analytical methods were validated and controlled as described in detail in previous articles (Brzostowski et al. 2009, 2011a, b). The statistical analyses were performed with the Statistica® program package (Statsoft, Inc.), using a nonparametric Mann-Whitney *U* test.

## Results and discussion

### Bioconcentration potential

The bioconcentration potential of a fungal species for the accumulation of elements is assessed using the bioconcentration factor (BCF) which is calculated as the quotient of concentration levels in the mushroom divided by the concentration in the topsoil (or other substrate). Depending on fungal species, its mycelium can penetrate into the surficial organic layer and also the deeper mineral soil, and determined BCF is always an estimated indication of elements uptake. In other words, the BCF relates to and assesses the potential of mycelia for the uptake of chemical element(s) from soil and soil solution and its accumulation in a fruiting body and its morphological parts (Brzostowski et al. 2011a). This idea is similar to the “dose-effect” concept by Paracelsus and relates to the total concentration of an element in a fruiting body (its chemical forms can be different depending on substrate, e.g., soil fractions and soil solution). Trace elements that are well absorbed by mycelium, both from geogenic and anthropogenic sources, include Ag, Cd, Cu, Hg, Rb, and Zn, and they can be very well bio-concentrated and reach high values of BCF (BCF > 1) (Falandysz and Danisiewicz 1995; Falandysz 2017; Li et al. 2016; Tyler 1982). Heavy metals (Cd, Hg, Pb, Ni) that originate from anthropogenic sources in polluted areas, may be well bio-concentrated or accumulated by some species while other elements are accumulated due to geogenic anomalies (Árvay et al. 2014; Barkan et al. 1998; Cejpková et al. 2016; Collin-Hansen et al. 2005; Kojta et al. 2015; Petkovšek and Pokorny, 2013). Other macro metallic elements that are in excess in soil substrata (e.g., Al, Ca, Fe) are excluded by the mycelia, and quantities accumulated in fruiting bodies are relatively low although they exceed the load of typical trace metallic elements (Ba, Co, Li, Ni, Sr). Pb, a widespread pollutant, also produces low BCF values in mushrooms (usually < 1) (Proskura et al. 2017).

The BCF value strongly depends on the determination method for the respective chemical element and its various chemical forms in soil and substrate. There are numerous methods, using different reagents, to determine the total, pseudo-total, or leachable content of a particular element (Cejpková et al. 2016; Davidson 2013; Lipka and Falandysz 2017; Kučak and Blanuša 1998; Proskura et al. 2017; Sastre et al. 2002). With the exception of Mn, the BCF values of the elements in *A. muscaria* were usually significantly higher for the labile than the “pseudo-total” fraction (Table 1). Mushrooms, both mycorrhizal and saprobic, are active participants in soil ecosystems; and through their filamentous structure, the hyphae are able to secrete organic acids and enzymes (Falandysz and Borovička 2013). They are able to modulate the pH in the vicinity of the hyphal filaments in order to better absorb inorganic compounds. The chelation and absorption features of a mushroom are species-specific and it is impossible to mimic them with chemical means.

Scarcity of some mineral nutrients or the requirement for another one may cause and accelerate co-absorption of other elements with similar chemical and physical characteristics although they are useless or toxic. Some elements can be co-associated for particular species or mushrooms from particular sites (e.g., Hg and Se) (Vogel-Mikus et al. 2016). Extensive studies from the northern hemisphere, where topsoil is usually slightly acidic, revealed that soil pH did not affect the bioconcentration and levels of Cd, Cu, Pb, and Zn (Gast et al. 1988) or Ag, Al, Ba, Ca, Cd, Co, Cr, Cu, Fe, Hg, K, Mg, Mn, Na, Ni, Sr, Pb, Rb, and Zn (Brzostowski et al. 2011a) in mushrooms. On the other hand, mushrooms may be able to adapt to small or geogenic concentrations of toxic elements, e.g., Ag, Cd, Hg, or Se via the formation of hardly soluble inorganic compounds (AgS, HgSe, HgS) and metalorganic complexes of Ag, Cd, Hg, and Se with firm bonds (Osobová et al. 2011; Schmitt and Meisch 1985; Vogel-Mikus et al. 2016).

Barium and strontium are similar chemically and physically to the essential element Ca, and all of them are weakly bioconcentrated (BCF around 1 or < 1) in mushrooms, e.g., (Brzostowski et al. 2011a); this is confirmed for our data on *A. muscaria* (Table 1). Barium and strontium in soil are in a form that is hardly soluble, and this can be one of the reasons for their weak bioconcentration by mushrooms. The same is the case for Ca which as a leachable fraction is relatively weakly bio-concentrated in caps and stipes of fruiting bodies. Its uptake can be in part dependent on co-occurrence of elements being in competition with Ca, such as Ba, Sr, and rare earth elements, or also due to possible chemical differences in Ca compounds in soil (Falandysz et al. 2017c). Ba and Sr from the highly weathered laterite read earths, red earths, and yellow earths of Yunnan in Asia, which are low in Ca, can be better bio-concentrated by mushrooms than from the European soils which are usually richer in Ca. Possibly, some of these species may have a preference for Ba and Sr from the laterite read earths, red earths, and yellow earths (Falandysz et al. 2017a, b; Saniewski et al. 2016). *A. muscaria* from the northern regions of Poland often bio-excluded Ba, Ca, and Sr by but also the metallic elements Ag, Al, Fe, Mn, and usually also Co (Table 1).

Fe and Al are both abundant in soils but commonly only minor in mushrooms (an exception for Fe is *Suillus variegatus*) (Drbal et al. 1975; Falandysz et al. 2001), and therefore, under typical environmental and soil conditions, these elements are bio-excluded by mushrooms. However, some doubts were raised about the accuracy of data reported earlier on Fe and Al in mushrooms (Borovička and Řanda, 2007).

*Amanita muscaria,* like all terrestrial mushrooms studied so far, was characterized by high BCF values for potassium (K). Fruiting bodies of mushrooms, e.g., *A. muscaria*, are physiologically rich in K, which is a major metallic cation (in this study median values of K in the caps were in the range of 36,000–53,000 mg kg^−1^ dm among the sites; Table 1). Also, parasitic mushrooms that develop rhizomorphs (mycelial cords), connecting soil, and infected plant, e.g., as is the case for some *Armillaria* species, are rich in K (48,000 ± 5700 mg kg^−1^ dm in the caps and 59,000 ± 40,000 mg kg^−1^ dm in the stipes) (Falandysz et al. 1992).

The median BCF values for leachable K by *A. muscaria* were in the range of 590–1400 for caps and 280–1200 for stipes, but substantially lower (in the ranges 82–150 and 34–110) for the “pseudo-total” fraction of K in soil. As expected, the values for hot extraction with concentrated (65%) nitric acid were much higher (medians in range 380–740 mg kg^−1^ dry matter, dm) than the values for cold-extraction with 20% nitric acid (medians in range 31–100 mg kg^−1^ dm) (Table 1).

The element Rb was even better bio-concentrated by *A. muscaria* than K, with an efficiency (median values of BCF) in the range of 670–2600 for caps and 110–1200 for stipes (leachable fraction), while the “pseudo-total” fraction was in the range of 130–220 for caps and 35–120 for stipes (Table 1). It is well known that Rb can be an abundant element in fruiting bodies of *A. muscaria* and in other mushrooms. For example, the median values of Rb were at 110 mg kg^−1^ dm in caps and 65 mg kg^−1^ dm in stipes of *A. muscaria* fruiting bodies (Drewnowska et al. 2013), and at 500 (21–1600) mg kg^−1^ dm in whole fruiting bodies of *Amanita rubescens* Pers. (Tyler 1982).

The fraction of magnesium in soils that was leachable with 20% nitric acid was easily taken up by *A. muscaria* and further bio-concentrated by its fruiting bodies in all the sites studied. The median BCF values calculated for leachable Mg in caps were in the range of 2.6–13 and for stipes in the range of 1.2–6.8. As expected, most of the Mg in the examined soils was in the pool of non-leachable, “pseudo-total” Mg (Table 1). “Pseudo-total” (and possible total) Mg, which is a geogenic metallic element in a top layer of forest soils, dominates and is less accessible for the mycelia. The median BCF values calculated for the “pseudo-total” Mg in *A. muscaria* from northern Poland were in the range of 0.65–2.7 for caps and in the range of 0.47–1.5 for stipes.

Among of the essential elements, the micro-constituents such as Cu and Zn were well bio-concentrated by *A. muscaria* in fruiting bodies, while less so for Na but still with a BCF > 1. The median BCF values for leachable Cu, Zn, and Na in caps were in the range, respectively, 53–160, 12–53, and 1.7–48, and for stipes were 18–79, 6.4–34, and 1.5–69 (Table 1). Also, the BCF values of Cu, Zn, and Na for the “pseudo-total” fraction were relatively high (usually > 1), i.e., the medians for caps and stipes respectively were in the ranges 18–130, 8.3–23, and 1.6–9.0 and 7.0–61, 5.3–13, and 0.98–40. This is discussed in detail below.

### Macro elements in mushrooms

#### Potassium

The median concentrations of K in *A. muscaria* caps ranged from 36,000–53,000 mg kg^−1^ dry matter (dm) and from 19,000 to 38,000 mg kg^−1^ dm in stipes (Table 1). The caps were substantially richer in K than the stipes—the median values of the Q_C/S_ index (which was calculated in the fruiting bodies as the quotient of metallic element concentration in caps divided by the concentration in the stipes) were in the range 1.7–1.9 for five sites and at 2.3 in the Giżycko site. Potassium in whole fruiting bodies of *A. muscaria* was at 33000 ± 6000 mg kg^−1^ dm in a study by Vetter (2005). Mushrooms from two sites (Dziemiany and Bydgoska forest) showed potassium in a substantially greater concentration than for the Pasym site (*p* < 0.05; Man-Whitney *U* test). The potassium concentration in fruiting bodies of *A. muscaria* correlated positively with cadmium (*r* = 0.61; *p* < 0.05) and cobalt (*r* = 0.65; *p* < 0.05).

The Dziemiany site was also characterized by greater K content in topsoil—both the “pseudo-total” and labile fractions, i.e., the median values were respectively at 74,000 and 11,000 mg kg^−1^ dm. Elsewhere in this study, the range of median values for the “pseudo-total” fraction was at 33–60, and for the labile fraction it was at 31–91 mg kg^−1^ dm.

As was mentioned, K was efficiently bio-concentrated by *A. muscaria* in fruiting bodies. The median values of BCF for the caps differed significantly for the sites and for the labile fraction were in the range 1200–1400 at the sites of Sobieszewo Island, Giżycko, and Dziemiany, and much lower for the three other sites which ranged from 590 to 820.

#### Magnesium

In all six sites, *A. muscaria* fruiting bodies contained Mg in similar (*p* > 0.05) concentrations (Table 1). Medians for caps were at 850–1100 mg kg^−1^ dm, with a total range of 580–1300 mg kg^−1^ dm, and for stipes the medians were at 440–600 mg kg^−1^ dm, with a total range of 300–680 mg kg^−1^ dm. The median values of the index Q_C/S_ for magnesium were in the range of 1.7–1.9 in five sites and 2.3 in the Giżycko site.

The sites differed in concentration levels of Mg in topsoil. The “pseudo-total” fraction of magnesium was at 400 to 1200 mg g^−1^ dm (medians) and the labile fraction was in the range of 92–310 mg g^−1^ dm. Magnesium was bio-included by *A. muscaria*, and the median values of BCF for this element in caps were in the range of 0.65–2.7 for the “pseudo-total” fraction and in the range of 2.6–13 for the labile fraction, and in stipes respectively at 0.47–1.5 and 1.2–6.8.

#### Rubidium

*Amanita muscaria* contained Rb in the caps in the range (medians) from 110 to 470 mg kg^−1^ dm, while for the stipes those values were substantially smaller and ranged from 66 to 310 mg kg^−1^ dm (Q_C/S_ index at 1.8–2.7 in five sites and 4.0 in the Pasym site).

Topsoil contained Rb in the “pseudo-total” fraction in the range (medians) from 1.2 to 4.4 mg kg^−1^ dm and in a much smaller portion in the labile fraction (range 0.27–1.0 mg kg^−1^ dm). The concentration levels of the labile fraction of Rb in forest topsoil were significantly lower at two sites: the Sobieszewo Island site, which is under impact by aerosol from the Baltic Sea and the most eastern location of this study in the Giżycko site (medians respectively at 0.27 and 0.27 mg kg^−1^ dm and in other sites at 0.66–1.0 mg kg^−1^ dm; *p* < 0.05). Rubidium was highly bio-concentrated in fruiting bodies of *A. muscaria*, and the median BCF values for the labile fraction were in the range of 670–2600. Due to the large difference between the contents of the labile and “pseudo-total” fraction of Rb in topsoil substrata, the BCF values for the “pseudo-total” fraction were much lower than for the labile fraction (Table 1).

#### Calcium

The median values of Ca concentration in the caps were in the range of 84–160 mg kg^−1^ dm and were at similar or greater level in the stipes in particular sites (range 77–260 mg kg^−1^ dm; Q_C/S_ index at 0.61–1.4). The woodland areas investigated differed significantly in content both of the “pseudo-total” and the labile fraction of calcium in topsoil. The Darżlubska Wilderness site was richer both in the “pseudo-total” and the labile fraction of Ca (medians respectively at 1400 and 1500 mg kg^−1^ dm) than were other locations. The “pseudo-total” fraction of Ca was in the range of 190–720 mg kg^−1^ dm in other sites. The difference in the content of the labile fraction of Ca in topsoil substrata between the sites was even greater than for the “pseudo-total” fraction, e.g., as mentioned, the median value was at 1500 mg kg^−1^ dm in the Darżlubska site, and for the other four sites it was in the range 230–690 mg kg^−1^ dm, while the lowest value of 41 mg kg^−1^ dm was found in the Giżycko site.

The median values of BCF for leachable Ca from caps and stipes of *A. muscaria* were respectively in a wide range: 0.17–2.2 and 0.16–2.3, and for “pseudo-total” Ca were 0.20–0.55 and 0.11–0.61. The BCF values calculated for the leachable fraction of Ca in caps and stipes of *A. muscaria* showed on inverse association with the content of the element in soil. Such an association indicates a tendency to maintain Ca homeostasis in the fruiting bodies.

Soils from the sites examined also highly differed in Ca content (*p* < 0.01; Mann-Whitney *U* test). The median concentration values for the leachable fraction of Ca was as low as 41 mg kg^−1^ dm for the Giżycko site and as high as 1500 mg kg^−1^ dm for the Darżlubska Wilderness site in the region nearby to the town of Wejherowo.

The median Ca values in caps were in the range from 84 mg kg^−1^ dm (Dziemiany site in the region of Kaszuby) to 160 mg kg^−1^ dm (Darżlubska Wilderness). Stipes tend to provide a better matrix for Ca than caps in mature fruiting bodies as Ca concentrations in stipes are typically greater than in caps (Kułdo et al. 2014; Kojta et al. 2016). In this study, the median values of the Q_C/S_ index for Ca were in the range of 0.61–1.0 at four sites (Darżlubska Wilderness, Bydgoskie forests, Dziemiany, and Pasym) and in the range of 1.1–1.4 at two sites (Giżycko and Sobieszewo Island).

#### Sodium

A half or more of Na determined in topsoil was in the leachable fraction for the study sites: the medians ranged from 5.8 to 24 mg kg^−1^ dm, while the “pseudo-total” fraction was in the range of 12–31 mg kg^−1^ dm. The Na concentrations in the caps of *A. muscaria* were half or lower than the levels in stipes, and the medians for the sites were respectively in the range of 31–110 mg kg^−1^ dm and 41–370 mg kg^−1^ dm. The BCF of Na in *A. muscaria* exceeded 1 for all locations, with median values in the range of 1.7–48 for caps and 1.5–69 for stipes (Table 1).

Edible mushrooms collected from the wild or cultivated are considered as low sodium food items (Vetter 2003).

### Trace elements

#### Manganese

The leachable fraction of Mn was relatively low in topsoil from the seaside region of Sobieszewo Island and also from the outskirts of the Giżycko site in the Great Lakes region of NE Poland, i.e., they were at 22 and 13 mg kg^−1^ dm (medians). Topsoil in other sites was richer in leachable Mn, with 44 mg kg^−1^ dm in the Bydgoska forests and in a range of 77–97 mg kg^−1^ dm for the other sites. Leachable Mn was at the same level as the “pseudo-total” fraction at four sites, while it was around 40% lower than the “pseudo-total” Mn in the Bydgoska forests and the Giżycko sites (Table 1).

*Amanita muscaria* bio-concentrated Mn with similar efficiency in caps and stipes of fruiting bodies. The median values of BCF for leachable Mn in caps were in the range of 0.13–1.0 and in stipes at 0.12–0.91, while for the “pseudo-total” fraction they were respectively in the ranges 0.13–0.56 and 0.11–0.51.

### Aluminum and Iron

Forest soils showed the leachable fraction of Al in the range of 1100–6700 mg kg^−1^ dm (medians) and of Fe at 620–1900 mg kg^−1^ dm, while the “pseudo-total” fractions were respectively in the range of 4000–27,000 mg Al kg^−1^ dm and 1200–3400 mg Fe kg^−1^ dm. With a high content of Al and Fe in soil substrata and under the typically slightly acidic pH conditions of forest soils from the northern regions of Poland, mycelia usually highly restrict their uptake of Al and Fe (Brzostowski et al. 2011a). The median values of BCF both for Al and Fe in *A. muscaria* were much lower than 1 (Table 1). Nevertheless, Al and Fe, with median contents in the caps in the range of 68–300 mg Al kg^−1^ dm and 65–230 mg Fe kg^−1^ dm, and in the stipes with 34–220 mg Al kg^−1^ dm and 54–110 mg Fe kg^−1^ dm, were at the levels of Na, Rb, or Zn and higher than those of Cu (Table 1).

### Copper and zinc

Leachable copper values, when based on chemical extraction in vitro*,* were the highest (around 80%) in topsoil from the Bydgoska forest site and at the lowest level (around 30%) at the Giżycko site. In four study sites, leachable and “pseudo-total” Cu concentrations were approximately equal (Table 1). Soils from the background areas of northern Poland in this study reflect the typical composition of the parent rock and are without known geochemical anomalies in the upper lithosphere, while soil types are diverse and display a mosaic-like pattern (www. 2017a). The situation is different in the region of the Świętokrzyskie Mountains in southcentral Poland and towards the south, where some minerals, the copper, zinc, lead, and iron ores are or were major resources (Brzezicha-Cirocka et al. 2016), as well as in southwestern Poland with a large Cu ore deposit in the Legnica region (in the European part of the Circum Pacific Mercuriferous Belt anomaly, and these deposits are enriched with associated elements (Gustin et al. 1999; www. 2017b).

The efficiency of *Amanita muscaria* Cu bioconcentration varied among the sites. The median BCF values for the leachable and “pseudo-total” fractions were respectively in the range of: 53–160 and 18–130 for caps, and 18–79 and 7.0–61 for stipes (Table 1). Leachable Cu median levels were in the range of 0.31–2.3 mg kg^−1^ dm and the “pseudo-total” at 0.52–4.4 mg kg^−1^ dm, while quantities sequestered in the fruiting bodies were in the range of 30–48 mg kg^−1^ dm for caps and 10–24 mg kg^−1^ dm for stipes.

Clearly, *A. muscaria* itself was a better extractor and regulator of Cu uptake and sequestration when compared to predictions based on the concept of the “leachable” fraction of Cu in soil that is extracted by diluted (20%) nitric acid (Table 1).

The requirement of *A. muscaria* for Zn is greater than for Cu. Median quantities of Zn determined in mushrooms were in the ranges of 89–180 mg kg^−1^ dm for caps and 52–130 mg kg^−1^ dm for stipes. In the three sites of Sobieszewo Island, Darżlubska Wilderness, and Pasym, almost 90% of Zn was in the leachable fraction (median concentration levels between 7.2–16 mg kg^−1^ dm for the leachable fraction and 8.0–15 mg kg^−1^ dm for the “pseudo-total”) while in smaller proportion for the other sites (median concentration levels between 2.6–8.3 mg kg^−1^ for leachable Zn and 6.2–14 mg kg^−1^ for the “pseudo-total”; Table 1). In this aspect, certain sites were statistically different (0.01 < *p* < 0.05). Hence, zinc was easier available for mushroom at the Sobieszewo Island, Darżlubska Wilderness, and Pasym sites—with median BCF in the range of 12–19 for caps and 6.4–11 for stipes, while less available at the Dziemiany, Bydgoska forests, and Giżycko sites—with median BCF in the range of 23–53 for caps and 11–34 for stipes (Table 1).

#### Cobalt

Topsoil Co concentrations were in the range of 0.23–1.2 mg kg^−1^ dm for the leachable fraction and slightly higher, i.e., in the range of 0.39–1.5 mg kg^−1^ dm for the “pseudo-total” fraction.

Cobalt in the lithosphere is associated with Fe and Mn and under oxidizing acidic conditions is considered as a relatively mobile metallic element, but its migration can be inhibited when adsorbed onto hydroxides of Fe and Mn (Kabata-Pendias and Pendias 1999). A high ratio of the labile to the “pseudo-total” fraction of Co in forest topsoils in this study suggested a high mobility. On the other hand, the concentration levels of Co in this study seem typical for sandy soils in Poland and were in the range of 0.1–1.2 mg kg^−1^ dm, while at 0.2–34 mg kg^−1^ dm in organic soils (mean 3 mg kg^−1^ dm), at 5.5–19 mg kg^−1^ dm (mean 10.8 mg kg^−1^ dm) in alluvial soils and at 4–29 mg kg^−1^ dm (mean 3 mg kg^−1^ dm) in clayey soils (Kabata-Pendias and Pendias 1999).

The median values of Co in caps were in the range of 0.15–0.44 mg kg^−1^ dm and in stipes were at 0.13–0.37 mg kg^−1^ dm, i.e., the distribution between the caps and stipes of the fruiting bodies was largely equal, while the concentration levels were relatively low. Vetter (2005) reported, as determined by inductively coupled plasma-mass spectrometry, on a greater content of Co in whole fruiting bodies of *A. muscaria* at 1.4 ± 1.3 mg kg^−1^ dm, and for six other *Amanita* species, the values ranged from a non-detectable level (< 0.05 mg kg^−1^ dm) to 1.8 ± 4.0 mg kg^−1^ dm in *A. phalloides* (Fr.) Link. *A. fulva* Fr., a species which was not studied by Vetter (2005), was collected from a forest of different soil parent material in Poland and contained Co in the caps in the range of 0.028 ± 0.001–0.098 ± 0.061 mg kg^−1^ dm and in the stipes at 0.050 ± 0.001–0.11 ± 0.08 mg kg^−1^ dm, as determined by inductively coupled plasma-mass spectroscopy with dynamic reactive cells (ICP-DRC-MS) (Falandysz et al. 2017a). *Cantharellus cibarius* Fr., sampled from several sites in Poland and examined by ICP-DRC-MS, contained the element in fruiting bodies at the median level of 0.56 mg kg^−1^ dm but Co was much lower, i.e., from 0.046 to 0.076 mg kg^−1^ dm in *Cantharellus tubaeformis* Fr. (Falandysz et al. 2017b). Hence, species-specific Co accumulation of fruiting bodies is likely to account for the differences found in mushroom species that grow under the same forest soil conditions.

*Amanita muscaria* bio-excluded Co at three sites (Darżlubska Wilderness, Dziemiany, and Giżycko) where the median values of BCF for the leachable fraction were < 1, while they were in the range of 1.2–4.8 for caps and 1.1–3.0 for stipes at the three other sites (Island of Sobieszewo, Bydgoska forests, and Pasym) (Table 1). In another study, *Amanita fulva* also bio-excluded Co (BCF around 0.05) (Falandysz et al. 2017a).

#### Barium and strontium

Barium and strontium were closely correlated in fruiting bodies of *A. muscaria* in this study (*r* = 0.82; *p* < 0.01). Both Ba and Sr positively correlated with Ca (*p* < 0.05; Student’s *t* test) in the examined *A. muscaria*. The median values of Ba contents were in the ranges of 0.51–1.6 mg kg^−1^ dm in caps and 0.40–1.6 mg kg^−1^ dm in stipes, and of Sr were in the range of 0.19–0.69 and 0.20–0.68 mg kg^−1^ dm (Table 1).

As mentioned, both Ba and Sr were characterized by very low BCF values for the caps and stipes (BCF much lower than 1). The median values of Ba content in soils were in the range of 34–120 and of Sr at 7.4–17 mg kg^−1^ dm in the leachable fraction while at 49–150 and 15–34 for the “pseudo-total” fraction.

### Toxic elements

#### Cadmium, mercury, and silver

The elements Cd, Hg, and Ag were minor constituents in forest topsoils from the background areas in this study, i.e., the median values for leachable Cd were in the range of 0.006–0.10 mg kg^−1^ dm and for the “pseudo-total” Cd at 0.013–0.086 mg kg^−1^ dm; total Hg was at 0.010–0.051 mg kg^−1^ dm, and the “pseudo-total” Ag was at 1.4–5.0 mg kg^−1^ dm (four sites) (Table 1).

As was mentioned, Cd and Hg were well bio-concentrated by *A. muscaria*, but Ag only weakly. The median values of BCF for leachable Cd ranged from 270 to 2700 (caps) and from 130 to 1200 (stipes). For total Hg, they were in the range of 17–51 (caps) and 10–28 (stipes). Hence, both for Cd and Hg, *A. muscaria* plays a role in their cycling between deeper strata and the surface soil in a forest ecosystem. As assessed from the data for the “pseudo-total” fraction, silver was rather weakly bio-concentrated by *A. muscaria* at some sites and was bio-excluded in others. The median value of BCF for Ag in caps was 1.8 at the Dziemiany site and was in the range of 0.49–0.87 elsewhere, while for stipes, it was in the range of 0.38–0.52 at all the sites with data (Table 1).

The elements Cd, Hg, and Ag are closely correlated and are strong chalcophiles. They form compounds in mushrooms via bonds with thiols in glutathione and metallothionein-like polypeptides (Cd, Ag, Hg), bonds with phosphoglycoprotein (Cd) where sulfur is absent, while some can occur as organometallic compounds such as methylmercury (MeHg), in thiols in larger organic molecule such as glutathione, cysteine, and thiol (-SH) rich proteins or in inorganic compounds, e.g., HgS, SeHg in the cytosol and its structures and in the cell walls in general (Fischer et al. 1995; Vogel-Mikus et al. 2016; Kavčič et al. 2018; Kruse and Lommel 1979; Osobová et al. 2011; Schmitt and Meisch 1985).

Some *Amanita* species from the section *Lepidella* bio-concentrate (hyperaccumulate) Ag with high efficiency, e.g., *Amanita strobiliformis* (Paulet ex Vittad.) Bertill. (Borovička et al. 2007). Mercury, as can be concluded from the studies performed so far on several *Amanita* species that grew under typical environmental conditions, was bio-concentrated by mycelium in fruiting bodies with similar efficiency in *A. muscaria*, *A. rubescens*, *A. fulva*, and *A. vaginata* (Bull.) Lam. (Drewnowska et al. 2014; Drewnowska et al. 2012a, b; Falandysz et al. 2007b; Falandysz and Drewnowska 2015; Lipka and Falandysz 2017; Nasr and Arp 2011; Nasr et al. 2011). Studies of *Amanita echinocephala* (Vittad.) Quél., *A. manginiana* Hariot et Patouillard, and *A. vaginata* from Yunnan soils with elevated geogenic Hg revealed a mercury accumulation at concentration levels in the range of 2.9–7.3 mg kg^−1^ dm (caps) and at 1.8–4.2 mg kg^−1^ dm (stipes) (Falandysz et al. 2016).

Both Cd and Pb form weaker bonds with biomolecules when compared to Ag, which is a highly reactive cation in contact with peptides or proteins. The Ag ion is highly toxic for unicellular organisms and lower trophic web aquatic biota but also in general for the proteins in cells. Some mushroom species accumulate Ag with high efficiency and have elevated Ag levels, but nothing is known of the associated risk for consumers of these mushrooms (Byrne and Tušek-Žnidarič 1990; Borovicka et al. 2010; Falandysz et al. 1994a, b).

## Conclusions

*Amanita muscaria* growing in soils with different levels of the geogenic metallic elements (Ag, Al, Ba, Ca, Co, Cu, Fe, Hg, K, Mg, Mn, Na, Rb, Sr, and Zn) showed signs of homeostatic accumulation in fruiting bodies of several of these elements, while Cd appeared to be accumulated at a rate dependent of the concentration level in the soil substrate. This species is an efficient bio-concentrator of K, Mg, Cd, Cu, Hg, Rb, and Zn and hence also contributes to the natural cycling of these metallic elements in forest ecosystems.
